# The Term Microbe

**Published:** 1891-02

**Authors:** 


### THE TERM MICROBE.

The various terms formerly used indiscriminately to denote those infinitesimal living organisms, discernible only by the use of powerful magnifying lenses, viz., *schizophites*, *micrococci*, *chroococci*, *microsphores*, *micro-organisms*, *desmo-bacteria*, *bacteria*, *bacteridia*, *bacilli*, etc., etc , have become merged by common consent, into the word *microbe*, from *micros*, small, and *bois*, life, thus signifying *diminutive* life, as proposed by Dr.

Charles Sedillot, the eminent Surgeon of Strasburg, in a lecture read before the French Academy of Sciences, as early as 1878, and now generally adopted by writers as expressions of living microscopic germs.

